# Proximal segmentation of the dorsal mesogastrium reveals new anatomical implications for laparoscopic surgery

**DOI:** 10.1038/srep16287

**Published:** 2015-11-06

**Authors:** Daxing Xie, Chun Gao, An Lu, Liang Liu, Chaoran Yu, Junbo Hu, Jianping Gong

**Affiliations:** 1 Department of GI Surgery, Tongji Hospital, Tongji Medical College, Huazhong University of Science and Technology, Wuhan, 430030, PR. China

## Abstract

Generally, the gold standard of radical surgery for gastrointestinal (GI) tumors is *en bloc* resection of primary lesions and their related tissues. For gastric cancer, the ideal surgical treatment should be D2 radical gastrectomy plus complete mesogastrium excision. Complete mesogastrium excision is rarely done or mentioned since little is known about the mesogastrium and its presence is still with controversy. Topographically, the “mesogastrium” refers to a peri-gastric structure composed of “fascia propria”, enveloping lymph nodes, blood vessels and adipose tissues, which by connecting to the stomach, suspends from the posterior abdominal wall. In this study, by employing video laparoscopy, a number of proximal segments of dorsal mesogastrium were found being extensively scattered around the pancreas. The structure of the mesogastrium was further identified intraoperatively and then confirmed both grossly and histologically after the operation. Our results demonstrated the existence of mesogastrium (gastric mesentery) and its architecture. We suggest for the first time a “Table model” to describe the relationship between the stomach and gastric mesenteries enveloped by fascia propria, which might provide an improvement in the surgical methods for excision of gastric cancer.

The gold standard of radical surgery for tumors is *en bloc* resection of primary lesions and related tissues^1^. Specifically, in case of gastrointestinal (GI) cancers, the ideal surgical treatment should be total resection of primary lesions and complete mesentery excision, with blood vessels ligated at their roots. Examples include total mesorectal excision (TME) in radical resection of rectal carcinoma^2^,^3^,^4^ and complete mesocolic excision (CME) in the radical resection of colon carcinoma^5^. In spite of these accepted and common procedures, descriptions and classification of the architecture of the gastric mesentery have been lacking. Surgeons who perform radical resections of gastric carcinomas have to look for blood vessels in adipose or connective tissues to perform lymphadenectomy^6^,^7^,^8^,^9^. The traditional D2 lymphadenectomy is associated with massive bleeding (over 100 ml) and poor harvest of lymph nodes (The number of lymph nodes removed varies greatly with different patients)^8^,^10^,^11^. This is especially true in obese patients.

Mobilization of D2 gastrectomy under laparoscopy used to enter a plane unintentionally with less bleeding. Despite the surgical plane was important for surgeons, no study has described and characterized its features. In this study, by using video laparoscopy, spaces or gaps with membranes on the each side were identifiable clearly as having “plane” or “layered” borders. Membranes on the gastric side were found to contain arterial blood vessels and lymph nodes and to be, in conjunction with the stomach, suspended from the posterior abdominal wall^6^. We further confirmed both grossly and by post-op histological examinations from more than a hundred radical laparoscopic resections. Our results suggested a new conceptualization of “gastric mesenteries” and revealed new insights into the components and structure of gastric mesenteries. These results, in turn, allowed us to map how the mesogastrium develops from its embryological stages to that in a mature adult.

## Results

The demographic features of 105 patients in the study are detailed in Table 1. The 6 architectural sections of the mesogastrium identified in this study were: (1) the left and (2) right gastroepiploic mesenteries; (3) the left and (4) right gastric mesenteries, (5) the posterior gastric mesenteries and (6) the short gastric mesenteries.

### Left gastroepiploic mesentery

**Figure 1:**
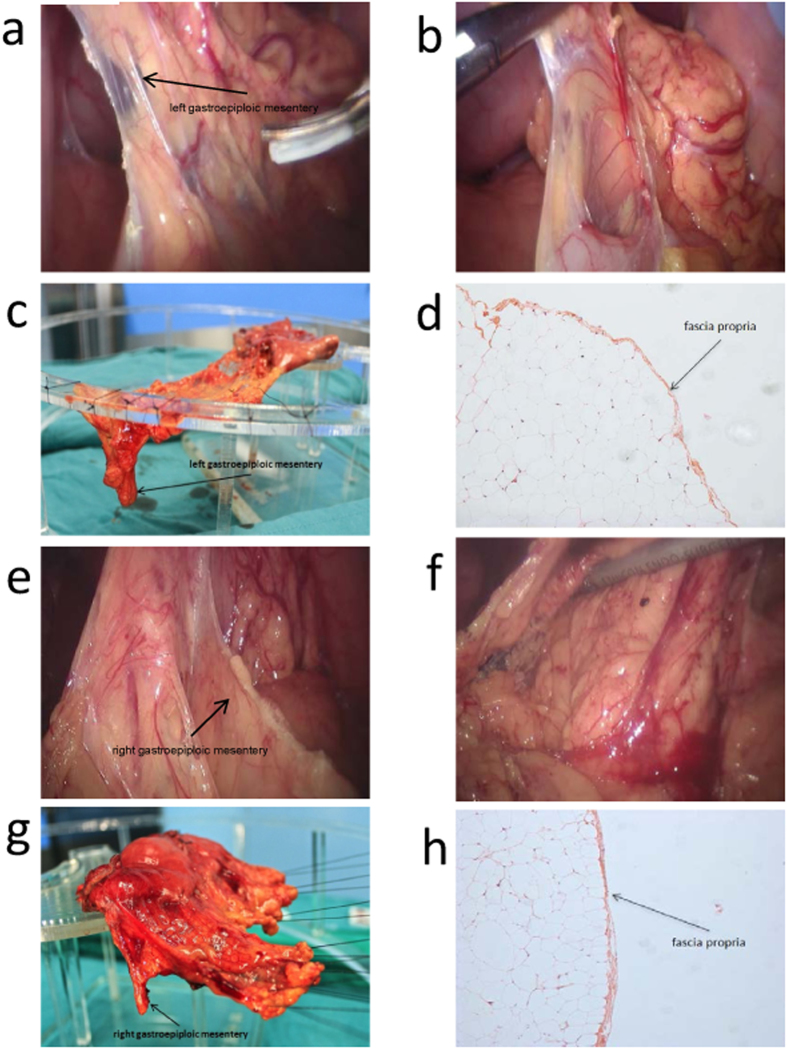
Left gastroepiploic mesentery and right gastroepiploic mesentery. Pictures of left gastroepiploic mesentery were photographed under laparoscopy before (**a**) and after dissection (**b**) during operation. The mesenteric surface could be observed. Left gastroepiploic mesentery was mounted on the stand before fixation (**c**) and photographed under the microscopy (**d**, HE 100 x). There exists fascia propria outside the adipose tissue of the mesentery. (**e–h**) shows the pictures of right gastroepiploic mesentery.

(Figure 1a–d) Cutting off the greater omentum from the left transverse colon and separation from the splenic flexure exposed the space containing Heald’s “angel hairs” (Holy Plane^12^) clearly revealing the fascia propria covering the smooth layer of the gastroepiploic mesentery on the surface of the mesocolon (Fig. 1a). We could see the smooth surface of the left gastroepiploic mesentery, which lay on the surface of the mesocolon and was covered by fascia propria (Fig. 1b). By using a home-made “endo-stripper”, the left gastroepiploic mesentery was stripped from the surface of transverse mesocolon all the way to border between the pancreas tail and the low hilus of spleen. The roots of the left gastroepiploic artery and vein were then separated and ligated. When the stomach and the greater mesentery were put on a stand, the dropping left gastroepiploic mesentery could be clearly seen (Fig. 1c). After photographing, the sample of the left mesogastrium was collected and fixed with formaldehyde solution.

By studying the slices made from the samples, we observed membrane structure outside the adipose tissues, i.e., the fascia propria under the microscopy (Fig. 1d). Because of the fascia propria, the adipose tissues covered by it presented a pumpkin-like surface (Fig. 1b).

### Right gastroepiploic mesentery

(Figure 1e–h) The membrane gaps were accessed from the aforementioned “tri-junction”, namely, a point where the mesentery of transverse colon, right gastroepiploic mesentery and the dorsal membrane of pancreas meet (Fig. 1e). Severance of the segments of the transverse colon attaching to the stomach revealed the “holy space”, where the right gastroepiploic mesentery was separated from the transverse mesocolon up to the roots of the right gastroepiploic vein and artery (Fig. 1f,g). After dissection of the lymph nodes 14v, the right gastroepiploic artery and vein were ligated at their roots. The light microscope also revealed a membrane namely a fascia propria (Fig. 1h) outside the adipose tissues.

### Left gastric mesentery

**Figure 2:**
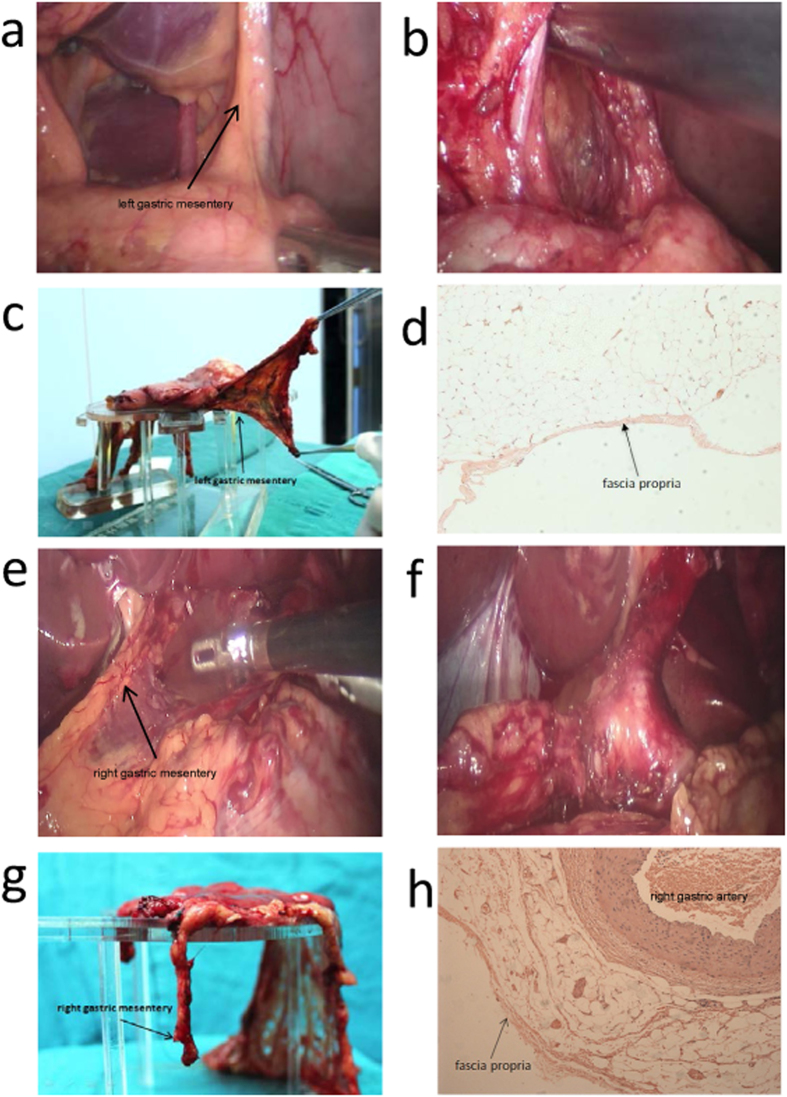
Left gastric mesentery and right gastric mesentery. Pictures of left gastric mesentery (**a–d**) and right gastric mesentery (**e–h**) were photographed similarly with Fig. 1. We fortunately observed the co-existence of right gastric artery and fascia propria (**h**).

(Figure 2a–d) Dissecting the serosa covering the pancreas and the common hepatic and spleen arteries and raising the lymph nodes 8a, 9 and 11p towards the right side revealed a space (Fig. 2a,b), defined by the left gastric artery on the right side and by the splenic artery and its mesenteric tissues on the lower side. The mesenteric tissue over the splenic artery was the left gastric mesentery. Pushing the left gastric mesentery upwards to the level of the upper part of the posterior stomach wall, along the abdominal aorta, to the crura of the diaphragm, expanded or enlarged this gap or space. After exposure of the left gastric artery and vein, they were ligated at their roots. The right side of left gastric mesentery was cut off at the right edge of the abdominal aorta and, on the left side, the mesentery was severed to the posterior gastric blood vessels.

### Right gastric mesentery

(Figure 2e–h) When the serosa that covers the gastroduodenal artery (GDA) was dissected, a space could be seen. After the gap was extended along the GDA, the common and proper hepatic arteries were revealed, lying under the lymph nodes 8a and the right gastric mesentery (Fig. 2e). After the lymph nodes 8a and hepatic artery proper were fully exposed, the right gastric artery and vein were ligated at their roots (Fig. 2f). With this sample, we were able to observe in this sample the co-existence of the right gastric artery and the fascia propria outside adipose tissues (Fig. 2g,h).

### Posterior gastric mesentery

**Figure 3:**
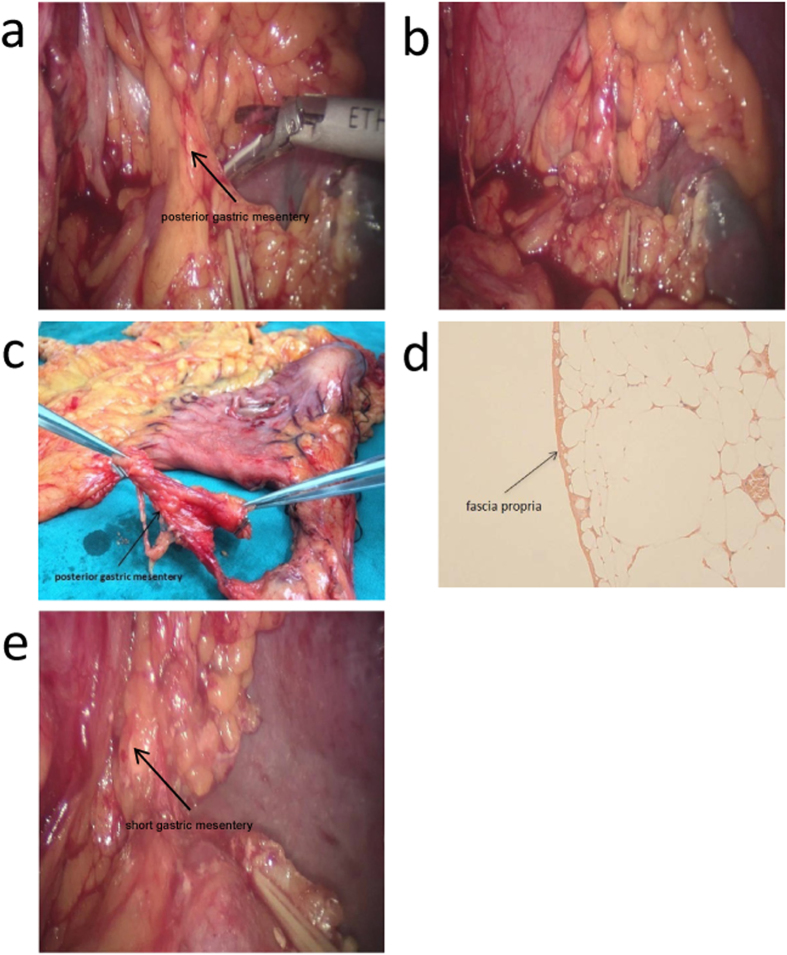
Posterior gastric mesentery and short gastric mesentery. (**a–d**) show posterior gastric mesentery. Two forceps were used to lift up the mesentery in (**c**), and we were able to observe the same structure of fascia propria in (**d**). (**e**) indicates short gastric mesentery.

(Figure 3a–d) Dissection of the left gastric mesentery and continued separation, postero-superiorly, along the splenic artery revealed a bundle of lustrous adipose tissues neighboring the left gastric mesentery and covered with membrane-like structures (Fig. 3a). This bundle of tissue was the posterior portion of the mesogastrium, through which the posterior gastric artery and a branch of the splenic artery runs. The posterior gastric mesentery extended to the crura of diaphragm. The posterior gastric mesentery was finally severed by ligating the posterior gastric artery and excising the veins at their roots (Fig. 3b). Again, we clearly observed the homogeneous fascia propria (Fig. 3c,d) outside the adipose tissues, which further verified and corroborated our perceptions of the structure of the mesogastrium.

### Short gastric mesentery

(Figure 3e) The mesentery was separated upwardly along the left gastroepiploic artery and then along the splenic vessels to expose the short gastric artery and vein. The two blood vessels, together with the adipose tissues and the fascia propria that surrounded them, form the short gastric mesentery (Fig. 3e). The short gastric mesentery extends upwardly to the left crura of diaphragm. To the naked eye, the short gastric mesentery was continuous with the left gastroepiploic mesentery. However, the two parts are separated by a fascia propria, a fact that has been repeatedly revealed during our surgical procedure. Due to its being short and small and susceptible to shrinkage when dissected, making it barely identifiable, we failed to harvest an actual specimen of this short gastric mesentery. Further research on the structure is now under way.

**Figure 4:**
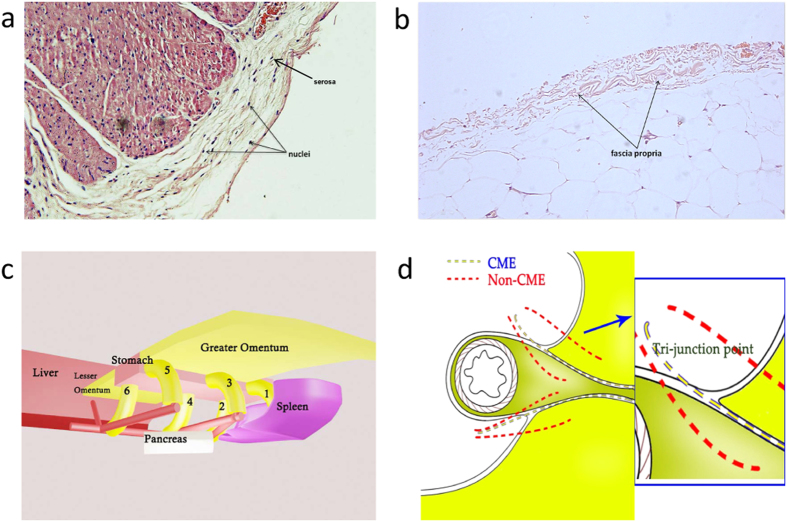
A proposed “Table Model”. The difference in the existence of mesothelial cells between serosa (**a**) and fascia propria (**b**). (**c**) shows the “Table Model” we put forward, and (**d**) indicates “Tri-junction” which is the access point of “D2 plus complete mesogastrium excision” procedure. ((**c**,**d**) were drew by Chaoran Yu).

It is worth noting that serosa differ from fascia propria in that there are mesothelial cells in serosa. Because of the presence of mesothelial cells (Fig. 4a) a number of nuclei could be seen in the serosa under a microscope. No nuclei were found in the fascia propria because the fascia propria contained no subcelluar structures and was simply a membrane structure comparable to collagenous fibers (Fig. 4b).

## Discussion

Through both gross histological observation and laparoscopic examination, this study has found and identified a hitherto unclassified anatomical architecture enveloping the stomach and surrounding adipose tissues, including lymph nodes and the blood vessels which help attach the structures to the posterior abdominal wall. Anatomically, these are precisely the essential features of a mesentery: connecting with an organ (stomach in this case), enveloping supplying blood vessels, lymph nodes and adipose tissues and suspending the organ onto the posterior abdominal wall. Different from the stereotypical image of mesentery, this “gastric mesentery” is not of sectorial shape^6^,^7^.

In order to describe the appearance and anatomic features of stomach and its mesenteries clearly, we put forward the notion of “Table Model” (Fig. 4c). Approximate shapes were used to represent certain organs or tissues, as the liver in the left part and spleen in the right part. The stomach is likened to a table in the central part, with the lesser omentum on its left upper side and the greater omentum on its right lower side. Numbers 1 to 6 represent the six portions of mesogastrium, which are: (1) short gastric mesentery, (2) posterior gastric mesentery, (3) left gastroepiploic mesentery, (4) left gastric mesentery, (5) right gastroepiploic mesentery, and (6) right gastric mesentery. The goal is to envision a situation under which the stomach was lifted up by laparoscopic clamps during surgery that looks like a flat table-top; the gastric mesenteries were therefore straightened and just look like six legs of this table. Though the six different separations of mesogastrium look isolated at the basal part, they are in fact anatomically connected at the top parts, together enveloping the stomach and forming a cavity surrounded by fascia propria, which provides space for the occurrence of “the fifth metastasis” of gastric cancer^6^.

**Figure 5:**
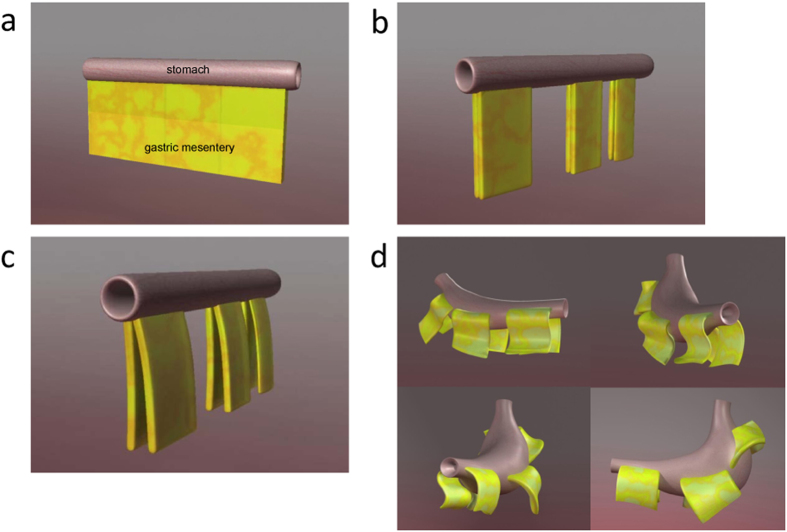
Development of stomach and mesogastrium. In (**a**), the stomach was still a tubular “proto-stomach” and its mesentery was continuous. In (**b,c**), the stomach began to expand and twist towards the left, and gastric mesentery was split into six sections. (**d**) indicates the process of clustering and coiling of gastric mesentery, and the mesentery were fused with the omentum as the last step.

The development, from a tube in the embryo to a fully-developed stomach in a mature adult, was proposed in our study. At its embryonic stage, the stomach is only a tubular “proto-stomach” and its mesentery is continuous and of sectorial shape (Fig. 5a). As it grows and develops, the stomach begins to expand and twist towards the left (Fig. 5b). At the same time, the pancreas and spleen began to grow larger. With these changes, the sectorial gastric mesentery is split at three sites, i.e., (1) the greater and lesser gastric mesentery, (2) edges of the greater or lesser curvature of stomach and (3) the end on the pancreas side (Fig. 5c). After the splitting, the gastric mesentery will “cluster” with the expansion or shrinkage of the stomach, forming 6 relatively independent regions. After “clustering”, the gastric mesentery will further “coil” downwardly onto the “mesenteric bed”, developing into a mature mesentery (Fig. 5d). We divided the coiled gastric mesentery into three parts: top, knee and base, respectively.

Here, it is necessary to mention the difference in the access point between complete mesogastrium excision (yellow line) and non-complete mesogastrium excision (red line) procedures. Complete mesogastrium excision gets access to the mesogastrium via the gap between middle adipose layer and two lateral adipose layers. The access point was dubbed “Tri-junction” (a junction of three adipose tissues running in different directions) which is the best entry point for complete mesogastrium excision (Fig. 4d).

Identifying the mesogastrium is significant for the standardization of radical surgery in gastric cancer. Standardization in radical gastric surgery procedures has long been advocated. In fact, it has been well established that the surgery starts from complete removal of the primary lesions and ends at the ligation of vessels at their roots (D2 lymphadenectomy), however, its boundary of resection has not been well defined. It was believed that the peri-gastric adipose and connective soft tissue have no definite borders. Our outline of the mesogastrium might now define their boundaries.

Establishment of the mesogastrial boundaries can facilitate the standardization of D2 radical surgery of gastric cancers, minimize the differences in the number of lymph nodes harvested, substantially reduce the intraoperative blood losses and surgery-related injuries, and diminish the possibility of “the fifth metastasis”^6^. For relatively obese patients, D2 operation can still be performed along the boundary.

In summary, this study describes the existence of gastric mesentery as identified by video laparoscopy and histological examination. Furthermore, we propose a “table model” to characterize the relationship between the stomach and gastric mesenteries. However, based on current observational stage, it is difficult to persuasively conclude that the existence of mesogastrium is omnipresent in every human being. Given the ethics restriction, healthy people and patients suffering from other diseases, theoretically reasonable for conclusive evidence and reducing the bias, are prohibited from being involved in the study. More evidences should be studied from adult cadavers. Nevertheless, we believe that the new conceptualization of the “mesogastrium” suggested by the study could present a new approach to improve mobilization of D2 gastrectomy under laparoscopy.

## Patients and Methods

### Patients

Between September 2011 to December 2013, 105 cases of advanced gastric cancer at stages of T2-T4a, N0-N2, M0 (NCCN’s TNM Staging) received laparoscopic D2 gastrectomy plus complete mesogastrium excision.

All procedures were performed under a protocol approved by the Ethics Committee at Tongji Hospital, and written informed consent was obtained from all patients. The methods were carried out in accordance with the approved guidelines by the Tongji Hospital Ethics Committee.

### Extent of lymphadenectomy

D2 lymphadenectomy was performed by following the guidelines stipulated in “the Japanese Classification of Gastric Carcinoma (English Edition, the 3^rd^ Edition)^13^. All the operation was performed by Prof. Jianping Gong, chief of general surgery of Tongji Hospital, Huazhong University of Science and Technology.

### Surgical techniques

Radical distal gastrectomy and D2 lymphadenectomy were performed using traditional methods modified in line with our concept of “partitioning (proximal segmentation) of the mesogastrium. After the great omentum was separated from the transverse colon, the posterior wall of stomach was raised from the surface of the pancreas. This way, the posterior wall of stomach and the major supplying blood vessels surrounded by adipose tissues were exposed. Held at this position, the stomach looks like a table-top and its supplying blood vessels are like the table legs. This procedure was therefore appropriately named the “table model”. It described and classified the different partitions of the mesogastrium and established the major arterial blood vessels in it. To ensure the complete removal of the mesogastrium, the intactness of the fascia propria should be guaranteed. Therefore, we first accessed the membrane gaps from a “tri-junction” we identified (to be detailed in the following section). Accordingly, the membrane gaps in the mesogastrium “tri-junction”, described in detail below in the “Results” section, were divided into 6 individually separate sections.

### Tissue sampling

Tissue samples were selected from patients with gastric cancer who had received total or distal gastrectomy^14^,^15^. This study is aiming to describe the existence of mesogastrium in human being. Therefore, mesogastrium tissues from those patients, including young and old, female and male, different tumor stages, are suggested to be enrolled and analyzed (Table 1). However, due to ethics issues, the mesogastrium of those healthy people and patients who suffer other diseases rather than gastric cancer are restricted.

The results were evaluated on a 5-point scale in terms of a wide array of measures, such as blood loss, completeness of mesogastrium, with “5” being the highest mark while “1” indicated the poorest sample (Table 2). Amongst cases, the complete mesogastrium which scored 4.5 or 5 were included in the sampling (Table 3). Immediately after surgical removal, the tissue specimens were mounted on a pre-fabricated stand for observing the mesogastrium and then photographed. In all cases, picture-taking was finished within 30 minutes after dissection. After photographing, the complete mesogastrium was aseptically removed from the sample by using surgical instruments and immediately fixed with 10% formaldehyde for 24–36 hours. After fixation, the samples were sent to the Department of Pathology, Tongji Hospital, for paraffin-embedding. The paraffin-embedded blocks were vertically sectioned, with each sample, into 5–10 slices, which were then mounted, HE-stained and then sealed.

The slices were then observed under a Leica DM16000B light microscope at different magnifications (50 x, 100 x, 200 x, 400 x, 630 x, *etc*), and images were obtained by employing the Leica Application Suite imaging software package. Meticulous examination of the gross sample and microscopic studies not only confirmed the existence or presence of the mesogastrium but also revealed its architecture.

For each portion of the mesogastrium, we took four pictures: before dissection, after dissection, before fixation (mounted on the stand), and a microscopic image, to present morphology and the components of the mesogastrium.

## Additional Information

**How to cite this article**: Xie, D. *et al.* Proximal segmentation of the dorsal mesogastrium reveals new anatomical implications for laparoscopic surgery. *Sci. Rep.* **5**, 16287; doi: 10.1038/srep16287 (2015).

## Figures and Tables

**Table 1:** Demographic features of 105 patients.

Variable	Data
Gender	
Male, n (%)	60 (57.14)
Female, n (%)	45 (42.8)
N (missing)	105 (0)
Pregnant	
Yes, n (%)	0 (0.00)
No, n (%)	45 (100.00)
Unknown, n (%)	0 (0.00)
Age (yr)	
Mean (SD)	52 (11)
Median	51
Min~Max	23~72
N (missing)	105 (0)
Height (cm)	
Mean (SD)	165.95 (6.34)
Media	167.00
Min~Max	155~178
N (missing)	96 (9)
Weight (kg)	
Mean (SD)	62.57 (10.26)
Media	62.5
Min~Max	44 ~ 95
N (missing)	99 (6)
Primary or recurrent tumor	
primary, n (%)	105 (100.00)
recurrence, n (%)	0 (0.00)
Tumor size	
Small, n (%)	<2 cm 18 (17.1)
Medium, n (%)	2–5 cm 78 (74.3)
Large, n (%)	>5 cm 9 (8.6)
Tumor depth (pT)	
pT2, n (%)	39 (37.1)
pT3, n (%)	45 (42.9)
pT4a, n (%)	21 (20.0)
Lymph node metastasis (pN)	
pN0, n (%)	24 (22.9)
pN1, n (%)	38 (36.2)
pN2, n (%)	28 (26.7)
pN3, n (%)	15 (14.2)
Preoperative treatment	
Yes, n (%)	0 (0)
No, n (%)	105 (100.00)

**Table 2:** Scoring of mesograstium.

	0 point	0.5 point	1 point
“Tri-junction”	fail to find	not obvious	very obvious
“Pumpkin-like surface”	fail to expose	not obvious	very obvious
“Little square”	fail to expose	not obvious	very obvious
Root ligation	fail to reach the root part of blood vessels	not quite satisfied	quite satisfied
Bleeding amount	>40 ml	20–40 ml	<20 ml

Five parameters were chosen mainly to evaluate the integrity of mesogastrium during surgery. “Tri-junction” and “pumpkin-like surface” can represent the locating and good expose of gastric mesenteries; “little square” refers to the flat and smooth plane on the lower side after gastric mesenteries have been well dissected, and root ligation means the dissection has reached the exit part of envolope-like mesogastrium. These four parameters together reflect the integrity of mesogastrium during and after surgical dissection. Besides, the bleeding amount in the laparoscopic part of surgery can tell the quality of operation as well.

**Table 3:** The point distribution of 105 cases in the study.

Score	Number of cases
5	19
4.5	27
4	29
3.5	17
3	9
2.5	3
2	1

Totally 46 cases got 4.5 or 5 points. There was one case of 2 points because of the rupture of right gastric artery during surgery.
